# Field-Induced Slow Magnetic Relaxation in Co^II^ Cyclopropane-1,1-dicarboxylates

**DOI:** 10.3390/molecules27196537

**Published:** 2022-10-03

**Authors:** Anna K. Matyukhina, Ekaterina N. Zorina-Tikhonova, Alexander S. Goloveshkin, Konstantin A. Babeshkin, Nikolay N. Efimov, Mikhail A. Kiskin, Igor L. Eremenko

**Affiliations:** 1 N.S. Kurnakov Institute of General and Inorganic Chemistry of the Russian Academy of Sciences, Leninsky Prosp. 31, 119991 Moscow, Russia; 2 A.N. Nesmeyanov Institute of Organoelement Compounds of the Russian Academy of Sciences, Vavilov St., 28, 119934 Moscow, Russia

**Keywords:** Co^II^ complexes, cyclopropane-1,1-dicarboxylic acid, dicarboxylate, coordination polymers, magnetic properties, single ion magnet, *SA-CASSCF/NEVPT2* calculations, *AILFT* calculations

## Abstract

New Co^II^ substituted malonate field-induced molecular magnets {[Rb_6_Co_3_(cpdc)_6_(H_2_O)_12_]∙6H_2_O}*_n_* (**1**) and [Cs_2_Co(cpdc)_2_(H_2_O)_6_]*_n_* (**2**) (where cpdc^2−^ stands for cyclopropane-1,1-dicarboxylic acid dianions) were synthesized. Both compounds contain mononuclear bischelate fragments {Co^II^(cpdc)_2_(H_2_O)_2_}^2^^−^ where the quasi-octahedral cobalt environment (CoO_6_) is complemented by water molecules in apical positions. The alkali metal atoms play the role of connectors between the bischelate fragments to form 3D and 2D polymeric structures for **1** and **2**, respectively. Analysis of dc magnetic data using the parametric Griffith Hamiltonian for high-spin Co^II^ supported by *ab initio* calculations revealed that both compounds have an easy axis of magnetic anisotropy. Compounds **1** and **2** exhibit slow magnetic relaxation under an external magnetic field (H*_DC_* = 1000 and 1500 Oe, respectively).

## 1. Introduction

The synthesis and study of paramagnetic complexes are of vital importance for achieving various goals, such as finding efficient magnetic refrigerators, elements for high-density data storage devices, and quantum computing [1,2,3,4,5,6]. Paramagnetic metal complexes that exhibit slow magnetic relaxation belong to the classes of single-molecule magnets (SMMs) or single-ion magnets (SIMs) [7]. A distinctive feature of SMMs is that exchange interactions exist in polynuclear molecules [8]. Usually, SIMs are mononuclear complexes in which the magnetic effects are determined by the magnetic anisotropy of the metal ion [9,10]. The properties of these compounds can be varied by changing the local geometry of the metal ion, the nature of the ligands, and the isolation of the paramagnetic centers in the crystal lattice. Currently, complexes of this kind are subject to studies in the field of spintronics, which is based on the effect of an external magnetic field on the spin states of a magnetic material [11,12].

3d-metal ions, for example, Fe^II^ or Co^II^, can exhibit magnetic anisotropy that depends on the geometry of the coordination environment [10,13,14,15,16,17,18]. The choice of 3d-metal undoubtedly affects the anisotropy and magnetic properties of the resulting coordination compounds [19,20,21]; however, selection of organic ligands that significantly affect both the structure of compounds and the possibility of exchanges between the metal centers is no less important. Dicarboxylic acids, for example, malonic acid and its substituted analogues, are versatile ligands since their anions can manifest both chelate and bridging coordination in binding metal atoms [22,23]. Varying substituents at the carbon atoms in acids and inorganic/organic cations makes it possible to expand the structural diversity of the resulting compounds [24,25,26]. The cobalt(II) atom in carboxylate complexes usually has an octahedral coordination environment, which leads to an unquenched orbital contribution to the *t_2g_* set and closely spaced excited states. Some of these compounds exhibit slow magnetic relaxation. Identification of relaxation paths is an important part of the analysis associated with magnetic data, supported by *ab initio* calculations.

Previously, the possibility to obtain field-induced SIMs on cobalt(II) malonates was shown for complexes with anions of benzylmalonic acid [27]. Here, we report two new polymeric cobalt(II) complexes with anions of cyclopropane-1,1-dicarboxylic acid and rubidium and cesium atoms. Both compounds showed slow relaxation of magnetization in the applied field.

## 2. Results and Discussion

### 2.1. Synthesis and Crystal Structure

The reaction of cobalt(II) acetate with M_2_cpdc (M = Rb, Cs) in water with heating results in the compounds {[Rb_6_Co_3_(cpdc)_6_(H_2_O)_12_]·6H_2_O}*_n_* (**1**) and [Cs_2_Co(cpdc)_2_(H_2_O)_6_]*_n_* (**2**). Compound **1** can also be prepared in a higher yield by refluxing the reaction mixture for 3 h in water.

In the IR spectra, we observe the preservation of asymmetric stretching vibrations and skeletal vibrations of the cyclopropane moiety: 3033 and 3031 cm^−1^ for **1** and **2**; and δ = 1042 and 1043 cm^−1^ for **1** and **2**, respectively. Moreover, the appearance of characteristic peaks of the COO^−^ groups is observed for complexes **1** and **2**: ν*_as_* = 1523 and 1526 cm^−1^; ν*_sym_* = 1402 and 1405 cm^−1^; and δ = 926 and 933 cm^−1^, respectively [28].

Complexes **1** and **2** crystallize in triclinic (group *P*-*1*) and monoclinic (group *P*2_1_/*c*) crystal systems, respectively. The structures of these compounds are based on the {Co^II^(cpdc)_2_(H_2_O)_2_}^2^^−^ bischelate moiety. Earlier, coordination compounds with dimethylmalonic acid anions in the cesium–cobalt(II) system were reported, in which the {Co^II^(R_2_mal)_2_}^2−^ bischelate moiety (where R_2_mal^2−^ is the dianion of the substituted malonic acid) was not formed [29].

The study of **1** and **2** by powder diffraction confirmed the conjunction with the structure of the corresponding single crystals and purity of the obtained powders (Supplementary Materials: S-1).

**Figure 1:**
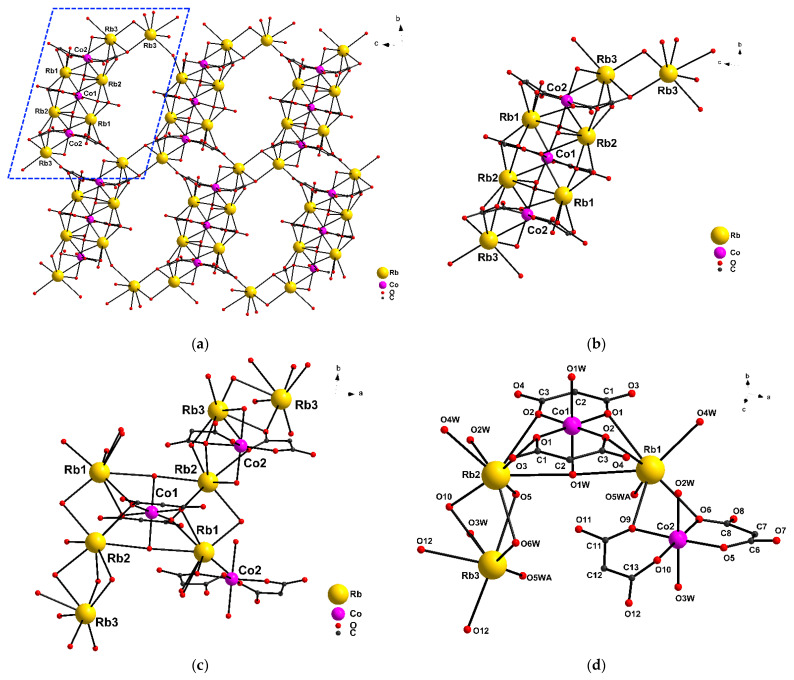
(**a**) The framework fragment of complex **1**; (**b**,**c**) the fragment of complex **1** along axis **a** and **c**, respectively; (**d**) a fragment of the independent part of the cell complex (hydrogen atoms, carbon substituents, and solvent molecules are hidden for clarity).

Compound {[Rb_6_Co_3_(cpdc)_6_(H_2_O)_12_]·6H_2_O}*_n_* (**1**) is a framework polymer (Figure 1a–d) in which the {Co(cpdc)_2_(H_2_O)_2_}^2^^−^ bischelate moieties are bound through the rubidium atoms. The complex contains two structurally non-equivalent cobalt(II) atoms, Co1 and Co2, each being located in a distorted octahedral environment, CoO_6_ (Figure 1d). The bischelate moiety {Co1(cpdc)_2_(H_2_O)_2_}^2^^−^ is centrosymmetric; the inversion center passes through the Co1 atom, and the cpdc^2−^ anion that forms a {Co1/O1/C1/C2/C3/O2} 6-membered ring exhibits a *κ*^2^,μ_5_-type coordination (Supplementary Materials, Figure S2.1), and is bound to the Rb1 and Rb2 atoms (Co1-O (cpdc^2^^−^) 2.029(3)-2.054(2) Å, Rb1-O1 2.980(2) Å, Rb1-O2 3.098(2) Å, Rb2-O1 (cpdc^2−^) 3.059(3) Å, Rb2-O2 3.156(3) Å). 

The {Co2(cpdc)_2_(H_2_O)_2_}^2−^ moiety occupies a general position. The cpdc^2−^ dianion also involved in the formation of the {Co2/O5/C6/C7/C8/O6} 6-membered ring has a *κ*^2^,μ_4_-type coordination mode and is bound to the all types of Rb atoms (Figure S2.1, Co2-O(cpdc^2−^) 2.027(3)-2.056(3) Å, Rb1-O6 2.884(3) Å, Rb2-O5 3.034(3) Å, Rb3-O5 3.222(3) Å), while the second cpdc^2^^−^ dianion in the {Co2/O9/C11/C12/C13/O10} cycle has a *κ*^2^,μ_5_-type coordination (Co2-O(cpdc^2^^−^) 2.036(2)-2.056(3) Å, Rb1-O9 2.895(3) Å, Rb2-O10 2.995(2) Å, Rb3-O10 2.979(2)Å, Rb3-O12 2.897(3)Å). Due to the large number of water molecules coordinated to rubidium atoms, a framework is formed (Figure 1a). The coordination spheres of the Co atoms are complemented by two µ_3_-H_2_O (Co1-O1W 2.127(2) Å) or two µ_2_-H_2_O (Co2-O(H_2_O) 2.138(3), 2.145(3) Å) molecules to form a distorted octahedral geometry (S_Q_(Co1) = 0.060, S_Q_(Co2) = 0.152) [30]. The Rb atoms in **1** are in three structurally non-equivalent positions. The coordination environment of the Rb atoms is formed by O atoms of the cpdc^2^^−^ dianion and water molecules and corresponds to a capped octahedron for Rb1 (RbO_7_), biaugmented trigonal prism for Rb2 (RbO_8_), and capped trigonal prism for Rb3 (RbO_7_) [30]. The main interatomic distances are indicated in Table S1. 

**Figure 2:**
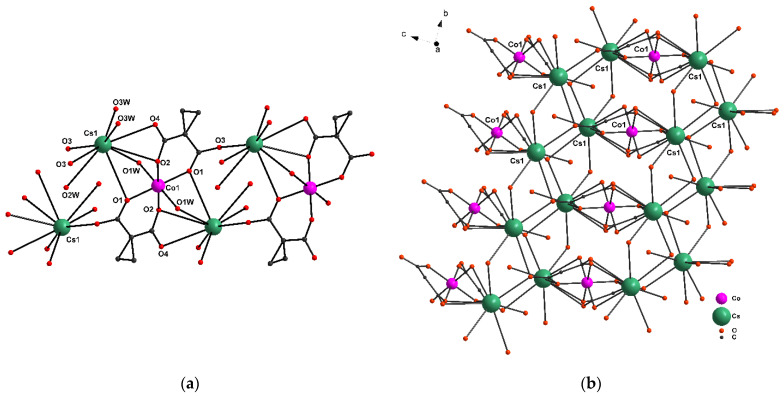
(**a**) A fragment of complex 2; (**b**) the layer fragment (hydrogen atoms and carbon substituents in (**b**) are hidden for clarity).

Replacement of Rb atoms with Cs leads to a decrease in the dimensionality of the polymer form from 3D to 2D. The {Co1(cpdc)_2_(H_2_O)_2_}^2^^−^ bischelate moiety is centrosymmetric; the inversion center coincides with the coordinates of the Co1 atom. The distorted octahedral environment of the Co atom (CoO_6_, S_Q_ = 0.109) in the symmetrical {Co^II^(H_2_O)_2_(cpdc)_2_}^2−^ moiety is formed by two cpcd^2−^ dianions that exhibit a *κ*^2^,μ_5_-type coordination and form {Co1/O1/C1/C2/C3/O2} 6-membered chelate rings, as well as by two bridging O atoms of water (Co-O(cpdc^2−^) 2.0510(16)-2.0550(15) Å, Co-O1W 2.1258(17) Å, Cs-O(cpdc^2−^) 3.1920(2)-3.5114(17) Å, Cs-O1W 3.2245(18) Å) (Figure 2a). The layer in **2** is formed by Cs atoms via carbonyl and O1W atoms of the bidentate water (Figure 2b). The Cs atoms have a coordination environment, which is formed by O atoms of cpdc^2−^ dianions and water molecules and corresponds to sphenocorona (CsO_9_) [30] (Cs-O(cpdc^2^^−^) 3.1920(2)-3.5114(17)Å, Cs-O(H_2_O) 3.0940(2)-3.3240(2)Å). The interatomic distances Co···Co for complexes **1** and **2** are 6.485 to 5.265 Å, respectively.

### 2.2. Magnetic Properties

#### 2.2.1. *Ab Initio* Calculation of the Electronic Structure and the Griffith Hamiltonian Approach

**Figure 3:**
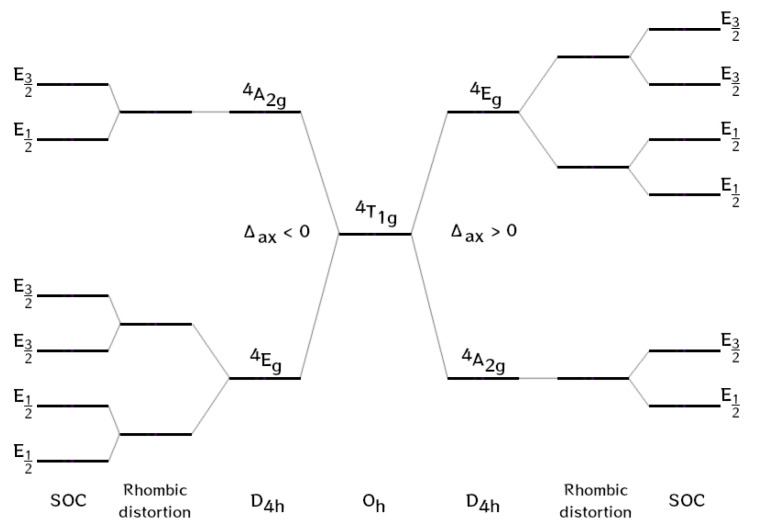
Energy level diagram for six-coordinate high-spin Co^II^ complexes; term splitting in spherical symmetry, splitting due to lowering to D_4h_ symmetry, and further splitting after inclusion of SOC (in a double-group notation).

It has been widely discussed that the ZFS spin-Hamiltonian (SH) is not always applicable to the description of pseudo-octahedral cobalt(II) complexes due to the significant contribution of the unquenched orbital angular momentum [31,32]. The ground term of the crystal field of the high-spin Co^2+^ ion in octahedral geometry is the 4T_1g_ orbital triplet (L = 1). The axial crystal field that arises due to distortion splits the 4T_1g_ term into an orbital doublet 4E_g_ and an orbital singlet 4A_2g_ (Figure 3). The SOC further splits the 4E_g_ term into four Kramers doublets (first-order effect) and also mixes 4E_g_ and 4A_2g_, resulting in the splitting of the 4A_2g_ term into two Kramers doublets (second-order effect) (Table 1). When the axial parameter Δ*_ax_* is positive, the ground term proves to be the orbital singlet 4A_2g_, while in the opposite case, when Δ*_ax_* is negative, the orbital doublet 4E_g_ becomes the ground state [27,33].

Using the *SA-CASSCF* method, it was shown by calculations that **1** and **2** are characterized by the presence of an “easy axis” of magnetization; that is, the orbital doublet proves to be the ground term in the axially distorted octahedron, while the rhombic crystal field splits the 4E_g_-term, thus resulting in tri-axial magnetic anisotropy [17,34]. This system can be described by the Griffith Hamiltonian (GH) equation, which takes both the crystal field (CF) and the Zeeman interaction into account:

(1) $$H=-\frac{3}{2}\kappa \lambda LS+\Delta_{ax}\left[L-\frac{1}{3}L\left(L+1\right)\right]+\Delta_{rh}\left({\hat{L}}_{X}^{2}-{\hat{L}}_{Y}^{2}\right)+\mu_{B}B\left(g_{e}\hat{S}-\frac{3}{2}\kappa L\right)$$

where *λ* is the spin-orbit coupling parameter, which typically ranges from –180 cm^−1^ to –130 cm^−1^; *κ* is the orbital reduction factor, which can vary from 0.6 to 1.0, depending on the complex; and; $\hat{L}$ and *Ŝ* are the orbital angular momentum and spin operator, respectively.

#### 2.2.2. Theoretical Modeling the Orbital Splitting

**Figure 4:**
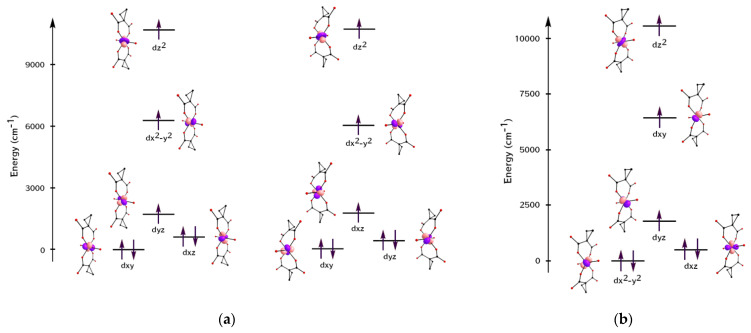
(**a**) Co^2+^ ion d-AO splitting in {Co1(cpdc)_2_(H_2_O)}^2−^ (left) and {Co2(cpdc)_2_(H_2_O)}^2−^ (right) anions in complex **1; (b)** Co^2+^ ion d-AO splitting in [Co1(cpdc)_2_(H_2_O)]^2−^ anion in complex **2**.

As expected for pseudo-octahedral complexes, two sets of split *t*_2g_ and *e*_g_ orbitals were found in **1** and **2**. The AO splitting diagram (Figure 4) was obtained from *AILFT* analysis [35]. The relative energies of the orbitals are given in the Table S2.

For the Co1^2+^ and Co2^2^^+^ ions of complex **1**, the splitting of the *d*-orbitals perfectly fits the D_4h_ octahedral geometry of the coordination environment. A similar arrangement of orbitals for cobalt(II) complexes with easy-axis magnetization types was described [36,37]. The splitting of *d*-orbitals for the Co^2+^ ion of complex **2** does not corresponds to D_4h_. The presence of cesium atoms in the structure of complex **2** causes strong destabilization of the *d*x^2^-y^2^-orbital (planar elongation) [38]. This is confirmed by the Co–O bond lengths (cpdc^2−^) (Table S1).

The calculated data obtained for complexes **1** and **2** are in good agreement with the typical values characteristic of cobalt complexes in a distorted octahedral environment (from −500 to −2000 cm^−1^) [36,37,39,40,41]. Since complex **1** contains two independent cobalt(II) ions with different degrees of the polyhedra deviation from the ideal geometry, it can be assumed that the largest contribution to the transition states is made by the Co2 atom. The total magnetization reversal barrier was higher than found for complex **2**. This can be explained by the larger Δ*_ax_*. Furthermore, the values of the remagnetization barrier in the applied field are characteristic of such quasi-octahedral complexes with high axial anisotropy [36,38,42]. The second factor that can be taken into account is a decrease in the dimensionality of the polymer and a lower isolation of the paramagnetic centers; in other words, shortening the Co∙∙∙Co distance, and thereby increasing the long-range magnetic dipole–dipole interactions and increasing the relaxation time of the magnetization [43,44].

#### 2.2.3. DC Magnetic Data

**Figure 5:**
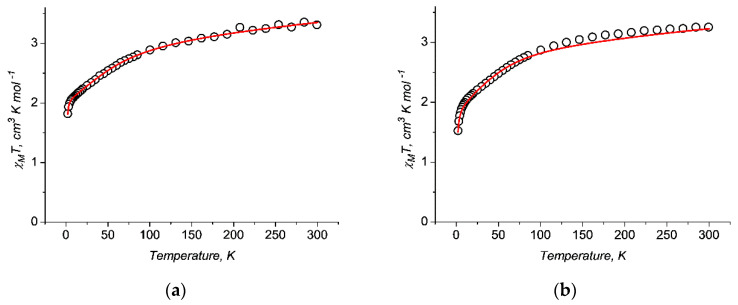
Temperature dependence of χ_M_T for **1** (**a**) and **2** (**b**) (calculated per one Co atom) measured at H = 5000 Oe. The lines show approximations by the Griffith Hamiltonian equation [31].

The magnetic properties of **1** and **2** were investigated in the temperature range of 2–300 K in an external magnetic field of 5000 Oe to obtain the χ_M_T(T) plots (Figure 5). For compounds in an octahedral environment, the χ_M_T values at 300 K (3.31 cm^3^·K·mol^−1^ and 3.26 cm^3^·K·mol^−1^ for **1** and **2**, respectively) significantly exceed the theoretical values for one magnetically isolated Co^2+^ ion (1.875 cm^3^·K·mol^−1^). This difference is most likely due to the large contribution of the spin-orbital moment to the total magnetic moment of the Co^2+^ ion. In both cases, the decrease in χ_M_T slowly accelerates with decreasing temperature, from 3.31 cm^3^ K/mol and 3.26 cm^3^·K·mol^−1^(at 300 K) to 1.82 cm^3^·K·mol^−1^ and 1.53 cm^3^·K·mol^−1^(at 2 K) for for **1** and **2**, respectively. In the case of Co^2+^ complexes, this magnetic behavior is caused by the anisotropy of the Co^2+^ ions and the Zeeman effect caused by the applied field [45,46].

These values were then fixed to fit the dc magnetic properties in order to reduce the number of varied parameters in the GH to only two, namely, *λ* and *κ* [31]. By simultaneous fitting of the temperature dependence (Figure 4), we obtained the best-fit values: average *λ* = −175.5 cm^−1^, *κ* = 0.87, χ*_tip_* = 1.84·10^−3^ cm^3^·K·mol^−1^, and zJ = 0 cm^−1^ for **1;** and *λ* = −175.4 cm^−1^, *κ* = 1.03, χ*_tip_* = 1.12·10^−3^ cm^3^·K·mol^−1^, and zJ = –0.051 cm^−1^ for **2**. The calculation for **2** takes into account weak intermolecular interactions, which may be due to the closer arrangement of cobalt atoms in the crystal lattice compared to **1**.

#### 2.2.4. AC Magnetic Data

**Figure 6:**
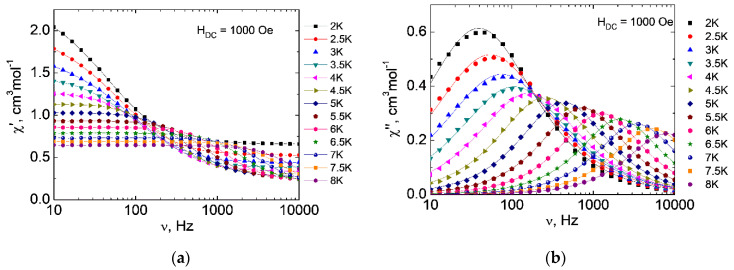
Frequency dependences of the real χ′ (**a**) and imaginary χ″ (**b**) components of the ac magnetic susceptibility of **1** in the 1000 Oe field (lines are guides for the eyes (χ′), approximation using the generalized Debye model (χ″)).

**Figure 7:**
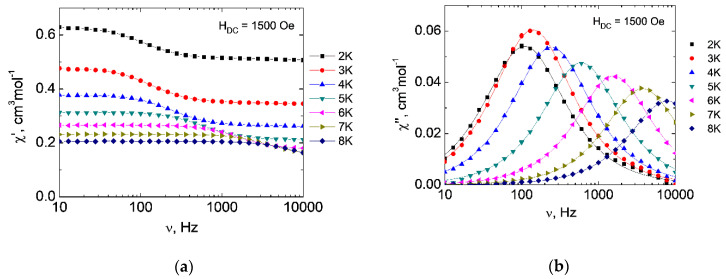
Frequency dependence of the real χ′ (**a**) and imaginary χ″ (**b**) components of the ac magnetic susceptibility of **2** in the 1500 Oe field (lines are guides for the eyes (χ′), approximation using the generalized Debye model (χ″)).

Recently, considerable attention has been paid to the slow magnetic relaxation of complexes of *d*-elements and especially Co^2+^; in other words, to their properties as molecular magnets [9,15,16,18,47]. In order to check the presence of slow magnetic relaxation for the complexes obtained, studies of the dynamic magnetic susceptibility of all the compounds were carried out (Figure 6 and Figure 7). As a result of these measurements, the frequency dependences of the real (in-phase, χ′) and imaginary (out-of-phase, χ″) components of magnetic susceptibility in magnetic fields from 0 to 5000 Oe at 2 K (Figures S3.1 and S3.2) were obtained.

In the zero dc magnetic field, no significant out-of-phase signals were observed for both complexes. In this case, deviations of the χ″(ν) dependences from zero are within the measurement error of the magnetometer. In order to reduce the possible effect of the quantum tunneling of magnetization (QTM), which can increase the relaxation rate significantly, subsequent measurements of the ac magnetic susceptibility of **1** and **2** were carried out in external magnetic fields of various strengths up to 5000 Oe.

**Figure 8:**
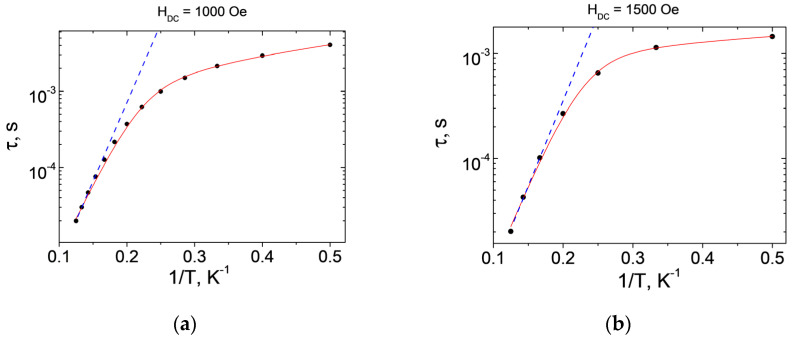
The τ(1/*T*) plots of the relaxation time of 1 (**a**) and 2 (**b**). Blue dashed lines—approximations of the high-temperature region using the Orbach relaxation mechanism; red solid lines—approximations using the sum of the Orbach and Raman mechanisms in the entire temperature range.

Applying an external magnetic field made it possible to study slow magnetic relaxation in **1** and **2**. The optimal dc magnetic fields corresponding to the largest values of the relaxation time are 1000 Oe for **1** and 1500 Oe for **2** (Figures S3.1 and 3.2). The frequency dependences of the in-phase and out-of-phase signals were obtained in the optimal magnetic fields (Figure 6 and Figure 7). According to the results of the approximation of isotherms χ″(ν) using the generalized Debye model, the dependences of the relaxation time on inverse temperature τ(1/*T*) were obtained (Figure 8).

In order to be able to compare the energy barrier height in the determination of the relaxation process parameters for compounds **1** and **2**, the high-temperature regions (Table 2) of the τ(1/*T*) plots were approximated by the Orbach relaxation mechanism τ_Or_^−1^ = τ_0_^−1^ exp(−Δ*E*/k_B_*T*), where ΔE is the height of the energy barrier of magnetization reversal, k_B_ is the Boltzmann constant, τ_0_ is the shortest relaxation time, and T is the temperature. The best-fit approximations were obtained using the sets of parameters for **1** and **2** presented in Table 2. The deviation from linearity (Figure 8) seen in the τ(1/*T*) plots in semi-logarithmic coordinates suggests that other than just Orbach mechanisms participate in magnetic relaxation.

The best-fit approximation of the τ(1/*T*) experimental data for complexes **1** and **2** was achieved using the sum of the Orbach and Raman relaxation mechanisms (τ^−1^ = τ_0_^−1^ exp(−ΔE/k_B_*T*) + C_Raman_T^*n*_Raman^), where C_Raman_ and *n*_Raman_ are parameters of the Raman relaxation mechanism. The best-fit results were found using the relaxation parameters presented in Table 2. The use of other mechanisms or sets of relaxation mechanisms results in values of parameters that are unacceptable from a physical point of view or in overparameterization. The two-phonon Raman process [48] dominates at low temperatures for both compounds.

The higher remagnetization barrier for **1** may be related to the higher axial component and lower rhombicity [33,36]. The second reason: the calculated first excited KD (268.9 and 237.9 cm^−1^) were higher for complex **1**.

## 3. Materials and Methods

### 3.1. Synthesis

#### 3.1.1. General Details

The new compounds were synthesized using distilled water and commercially available cobalt(II) acetate tetrahydrate (99%, Chempur, Karlsruhe, German), cyclopropane-1,1-dicarboxylic acid (97%, Sigma-Aldrich, Shanghai, China), rubidium hydroxide hydrate (Sigma-Aldrich, Shanghai, China), and cesium hydroxide monohydrate (Acros Organics, Great Britain). IR spectra of the complexes were recorded on a Perkin Elmer Spectrum 65 instrument using the ATR method in the frequency range of 4000–400 cm^−1^. Elemental analysis of the resulting compounds was carried out with an EA-3000 CHNS-analyzer (EuroVector, Pavia, Italy).

#### 3.1.2. Synthesis of New Compounds

{[Rb_6_Co_3_(cpdc)_6_(H_2_O)_12_]·6H_2_O}*_n_* (**1**).

*Method 1*. Co(OAc)_2_·4H_2_O (0.05 g, 0.20 mmol) was added to a solution of Rb_2_cpdc (obtained from RbOH∙xH_2_O (0.10 g, 0.80 mmol) and H_2_cpdc (0.05 g, 0.40 mmol)) in H_2_O (30 mL). The reaction mixture was stirred for 1 h with weak heating (*T* = 55 °C). The crimson solution thus obtained was slowly concentrated in an Erlenmeyer flask in air at room temperature within one month. The resulting crimson crystals were suitable for X-ray diffraction analysis. The yield of **1** was 0.05 g (42% counting per Co). 

*Method 2.* A mixture of RbOH∙xH_2_O (1.93 g, 16.08 mmol), H_2_cpdc (1.05 g, 8.04 mmol), and Co(OAc)_2_·4H_2_O (0.50 g, 2.01 mmol) in water (50 mL) was refluxed for 3 h in an oil bath. The crimson solution thus obtained was slowly concentrated in an Erlenmeyer flask in air at room temperature within one week. The resulting crimson crystals were suitable for X-ray diffraction analysis. The yield of **1** was 1.04 g (87% counting per Co).

Calc. (%) for C₃₀H₆₀Co₃O₄₂Rb₆: C 20.22; H 3.39. Found (%): C 20.41; H 3.62. IR spectra, ν/cm^−1^ (s = strong, m = middle, w = weak): 3264 m, 3033 m, 2284 w, 1523 s, 1434 s, 1402 s, 1241 m, 1208 s, 1078 w, 1042 w, 970 m, 926 m, 879 m, 863 m, 727 s, 614 s, 540 s, 454 s, 412 s.

[Cs_2_Co(cpdc)_2_(H_2_O)_6_]*_n_* (**2**).

Co(OAc)_2_·4H_2_O (0.10 g, 0.40 mmol) was added to a solution of Cs_2_cpdc (obtained from CsOH∙H_2_O (0.40 g, 2.40 mmol) and H_2_cpdc (0.16 g, 1.20 mmol)) in H_2_O (30 mL). The reaction mixture was stirred for 1 h with weak heating (T = 55 °C). The crimson solution thus obtained was slowly concentrated in air at room temperature within two weeks. The resulting crimson crystals were suitable for X-ray diffraction analysis. The yield of **2** was 0.19 g (70% counting per Co). Calc. (%) for C_10_H_20_CoCs_2_O_14_: C 17.43; H 2.93. Found (%) C 17.62; H 3.04. IR spectra, ν/cm^−1^: 3232 m, 3031 s, 1661 w, 1526 s, 1428 m, 1405 s, 1377 s, 1239 m, 1213 m, 1186 m, 1076 m, 1043 m, 968 m, 933 m, 877 m, 802 m, 740 s, 586 s, 542 s, 446 m, 435 m, 420 m.

### 3.2. Single-Crystal X-ray Diffraction Analysis

X-ray diffraction analysis was carried out on a Bruker Apex-II CCD diffractometer (graphite monochromator, *λ* = 0.71073 Å). An absorption correction was applied empirically using the SADABS program [49]. All the structures were solved in the OLEX2 and SHELXT programs [50,51] using the Intrinsic Phasing method and refined with SHELXL [52] using Least Squares refinement on F^2^. Nonhydrogen atoms were refined in anisotropic approximation. The hydrogens atoms of the methylene and water moieties were calculated according to the idealized geometry and refined with constraints applied to the C–H and O–H bond lengths and equivalent displacement parameters (U_eq_(H) = 1.2U_eq_(C); U_eq_(H) = 1.5U_eq_(O)).

The crystallographic parameters and refinement statistics are given in Supplementary Materials: Table S3. CCDC numbers 2161285 (for **1**) and 2161284 (for **2**) contain the supplementary crystallographic data for the reported compounds. These data can be obtained free of charge from The Cambridge Crystallographic Data Centre.

### 3.3. Powder X-ray Diffraction

The powder patterns of **1** and **2** were measured on Bruker D8 Advance (Bragg–Brentano geometry; sample dispersed thinly on a zero-background Si sample holder; CuKa radiation *λ* = 1.5418 Å (Ni filter); and θ/θ scan with variable slits) and Bruker D8 Advance Vario (transmission mode; sample deposited between two mylar films; CuKa1 radiation *λ* = 1.5406 Å (Ge monochromator); and θ/2θ scan) diffractometers, respectively, with a LynxEye detector from 5° to 60° 2θ, with a step size of 0.020°. The powder patterns Rietveld refinement was performed in TOPAS 5 software.

### 3.4. Magnetic Measurements

The magnetic properties of complexes **1** and **2** were investigated by dc and ac magnetic susceptibility measurements on a Quantum Design PPMS-9 magnetometer. The dc measurements were performed in the temperature range of 2–300 K in a constant external magnetic field of 5000 Oe. For the ac measurements, alternating fields of 5, 3, and 1 Oe in the frequency ranges 10–100, 100–1000, and 10–1,0000 Hz, respectively, were used. This procedure makes it possible to prevent overheating of the samples at low temperatures and produce the best signal-to-noise ratio. All magnetic behavior studies were carried out on milled polycrystalline samples sealed in plastic bags and frozen in mineral oil to prevent crystallite orientation in a magnetic field. The paramagnetic component of magnetic susceptibility (χ) was determined taking into account both the diamagnetic contribution of the sample itself, estimated from the Pascal constant, and the diamagnetic contributions of the mineral oil and the holder.

### 3.5. Computational Details

*Ab initio* (post Hartree–Fock) calculations of the zero-field splitting (ZFS) parameters and the g-tensor were performed based on the state-averaged complete-active-space self-consistent-field (*SA-CASSCF*) wave function [53] complemented by the N-electron valence second-order perturbation theory (*NEVPT2*) [54], using the ORCA program package (version 5.0.1) [55]. The calculations were performed with the geometry of the experimentally determined X-ray structures. The active space of the *CASSCF* calculations was composed of seven electrons in five d-orbitals of Co^2+^ ions (S = 3/2): CAS(7,5). The state-averaged approach was used, in which all 10 quartet (S = 3/2) and 40 doublet (S = 1/2) states were averaged with equal weights. The polarized triple-ӡ-quality def2-TZVP basis set was used for all the atoms [56]. An auxiliary def2/JK Coulomb fitting basis set was used in the calculation [57].

Both the zero-field splitting parameter (*D*) and transverse anisotropy (*E*), based on dominant spin-orbit coupling contributions from excited states, were calculated through the quasi-degenerate perturbation theory (*QDPT*) [58], in which approximation to the Breit–Pauli form of the spin-orbit coupling operator (*SOMF*) [59] and an effective Hamiltonian approach [60] were applied. The splitting of the d-orbitals was analysed within the *ab initio* ligand field theory (*AILFT*) [35,61].

## 4. Conclusions

New coordination compounds of cobalt(II) with anions of cyclopropane-1,1-dicarboxylic acid were obtained. In these complexes, the mononuclear fragments {Co^II^(cpdc)_2_(H_2_O)}^2^^−^ are bound by rubidium or cesium atoms to form 3D and 2D polymers, respectively. Both compounds demonstrate slow magnetic relaxation in a non-zero field (*H*_DC_ = 1000 and 1500 Oe). *Ab initio* calculations indicated the presence of an easy axis of magnetization in both complexes with a negative axial crystal field. Analysis of the magnetization relaxation mechanisms for complexes **1** and **2** suggest that a sum of the Orbach and Raman relaxation mechanisms operates. This may be implemented through under-barrier relaxation mechanisms.

## Figures and Tables

**Table 1:** SA-CASSCF-calculated spin-free and spin-orbit state energies (cm^−1^) for **1** and **2**.

Complex	Metal	Term	Energy Levels	
			Spin-Free State	Spin-Orbit State
**1**	**Co1**	4E_g_	0	0 268.9
			155.6	594.0 925.0
		4A_2g_	1715.4	2118.0 2216.1
	Co2	4E_g_	0	0 237.9
			354.6	688.2 988.2
		4A_2g_	1666.0	2008.3 2109.3
**2**	Co1	4E_g_	0	0 186.4
			678.2	895.7 1143.3
		4A_2g_	1742.8	2015.6 2107.6

**Table 2:** Approximation of the τ(1/*T*) plots for complexes **1** and **2** using various relaxation mechanisms.

Complex		1	2
**Optimal dc Field (Oe)**		1000	1500
Orbach	Temperature range (K) ΔE/k_B_ (K) τ_0_ (s)	6–8 48 5.2·10^−8^	7–8 37 2.0·10^−7^
Orbach + Raman	Temperature range (K) C (K^−*n*_Raman^ s^−1^) *n*__Raman_ ΔE/k_B_ (K) τ_0_ (s)	2–8 82 1.58 42 1.2·10^−7^	2–8 466 0.56 36 2.6·10^−7^

## Data Availability

The data presented in this study are available in this article and Supplementary Materials.

## Supplementary Materials

The following supporting information can be downloaded at: https://www.mdpi.com/article/10.3390/molecules27196537/s1, Figures S1.1 and S1.2: Powder X-ray diffraction data; Figures S2.1 and S2.2: Coordination modes of the cyclopropane-1,1-dicarboxylate dianions; Figures S3.1 and S3.2: AC data; Table S1: Interatomic distances and angles; Table S2: Relative energies of d-AO for **1** and **2**; Table S3: Crystal data and structure refinement for **1** and **2**; Table S4: H-bonds.

## Sample Availability

Samples of the compounds are available from the authors.
