# Supplementary material for: Field-Induced Slow Magnetic Relaxation in CoII Cyclopropane-1,1-dicarboxylates
Source: Molecules. 2022 Oct 3;27(19):6537. doi: 10.3390/molecules27196537 (PMC9572064; doi:10.3390/molecules27196537)
Supplement: Supplementary file 1 [file molecules-27-06537-s001.zip › molecules-1931666-supplementary.pdf]

Supplementary Materials

Field-Induced Slow Magnetic Relaxation in Co^{II} Cyclopropane-1,1-dicarboxylates

Anna K. Matyukhina [1], Ekaterina N. Zorina-Tikhonova [1,*], Alexander S. Goloveshkin [2], Konstantin A. Babeshkin [1], Nikolay N. Efimov [1], Mikhail A. Kiskin [1] and Igor L. Eremenko [1]

[1] N.S. Kurnakov Institute of General and Inorganic Chemistry of the Russian Academy of Sciences, Leninsky Prosp. 31, 119991 Moscow, Russia

[2] A.N. Nesmeyanov Institute of Organoelement Compounds of the Russian Academy of Sciences, Vavilov St., 28, 119934 Moscow, Russia

* Correspondence: ezorinatikhonova@gmail.com

Table of contents

S-1. Powder X-Ray diffraction data

S-2. Coordination modes of the cyclopropane-1,1-dicarboxylate dianions

S-3. Interatomic distances and angles

S-4. AC data

S-5. AILFT

S-6. Single crystal X-Ray diffraction data

S-7. H-bonds for 1 and 2.

S-1. Powder X-Ray diffraction data

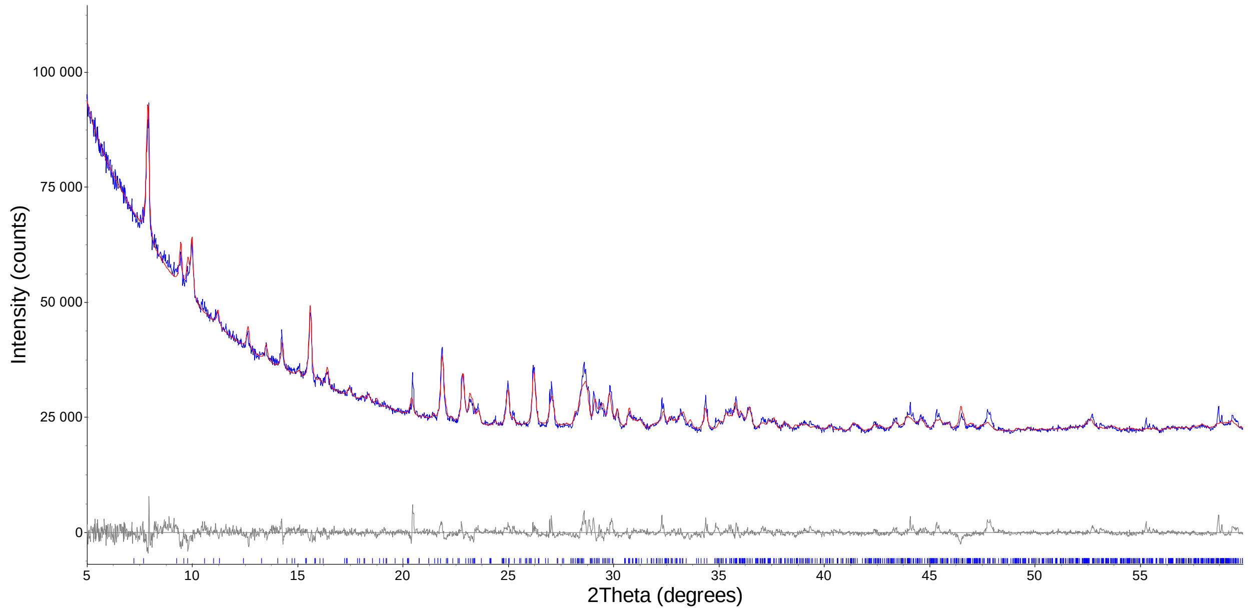

Figure S1.1. The experimental (blue line) and calculated (red line) powder patterns for compound **1** and their difference (grey curve). Blue ticks indicated calculated positions of the peaks.

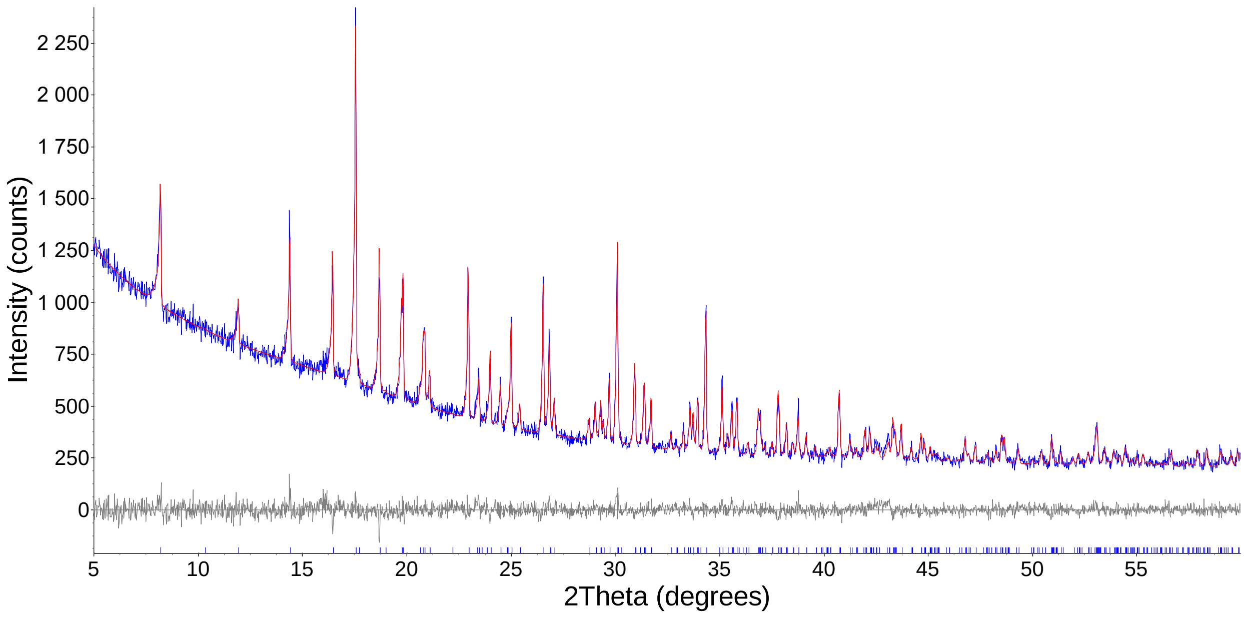

Figure S1.2. The experimental (blue line) and calculated (red line) powder patterns for compound **2** and their difference (grey curve). Blue ticks indicated calculated positions of the peaks.

S-2. Coordination modes of the cyclopropane-1,1-dicarboxylate dianions

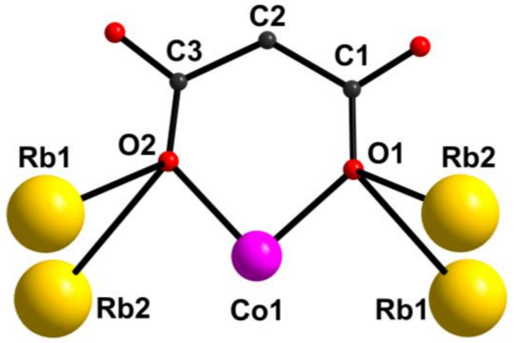

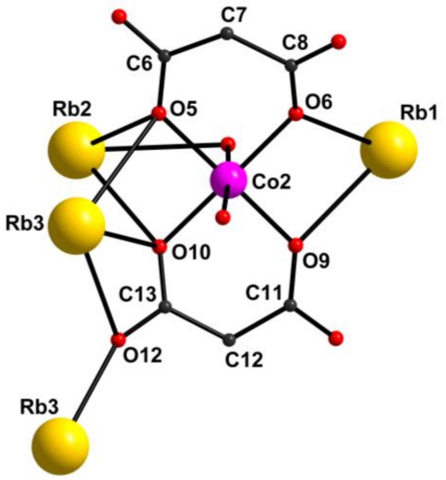

a b

Figure S2.1. Coordination modes of the cyclopropane-1,1-dicarboxylate dianions for **1**

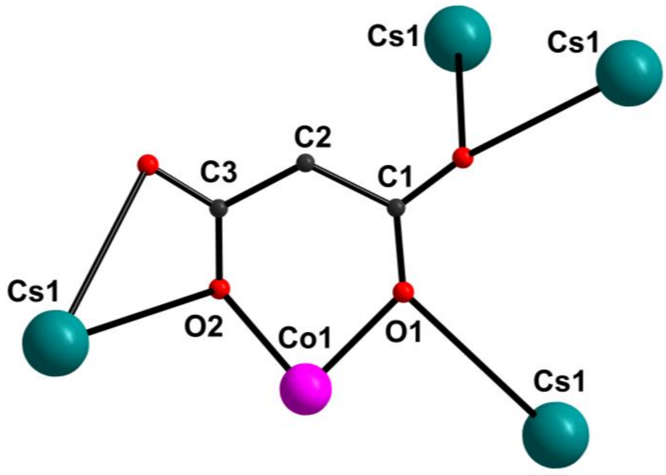

Figure S2.2. Coordination modes of the cyclopropane-1,1-dicarboxylate dianions for **2**

S-3. Interatomic distances and angles

Table S1. Selected interatomic distances [Å] and angles [°] for **1** and **2**

**Complex**	**1**	**2**
Co-O ($cpdc^{2-}$)	2.027(3)–2.056(3)	2.0510(16)–2.0550(15)
Co-O (H_2O)	2.127(2)–2.145(3)	2.1258(17)
M^I-O ($cpdc^{2-}$)	2.884(3)–3.222(3)	3.1920(2)–3.5114(17)
M^I-O (H_2O)	2.729(19)–3.203(3)	3.0940(2)–3.3240(2)
O-Co-O	87.18(10)–88.15(10)	86.74(6)
Co···Co	6.4854(6)	5.2648(2)
Co···M^I	3.6875(8)	4.0774(2)
M^I···M^I	4.0960(5)	5.2648(3)

S-4. AC-data

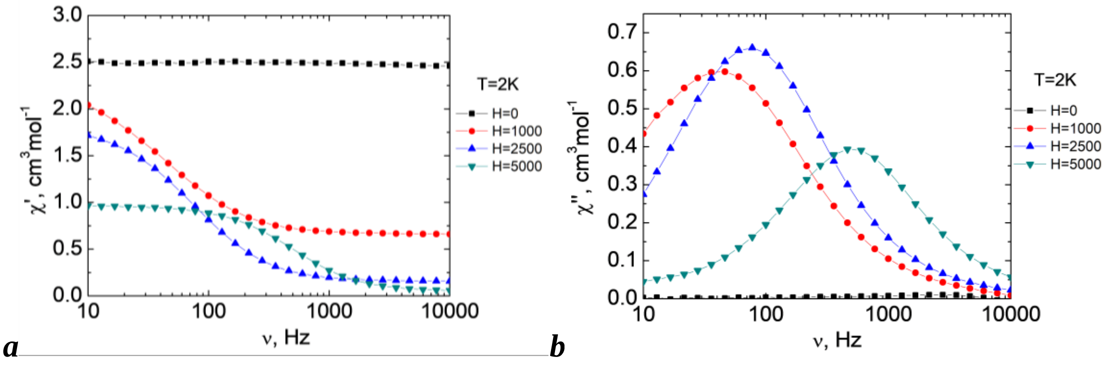

Figure S3.1. Frequency dependencies of real, χ' (***a***) and imaginary, χ'' (***b***) components of dynamic magnetic susceptibility for complex **1** at T = 2 K under various dc magnetic fields. Solid lines are visual guides.

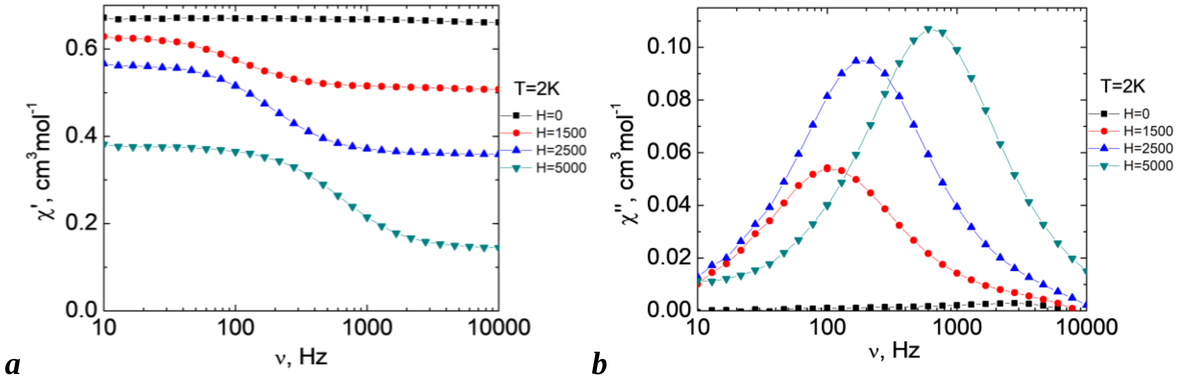

Figure S3.2. Frequency dependencies of real, χ' (***a***) and imaginary, χ'' (***b***) components of dynamic magnetic susceptibility for complex **2** at T = 2 K under various dc magnetic fields. Solid lines are visual guides.

S-5. Ab initio ligand field theory

Table S2. Relative energies of d-AO in complexes **1** and **2**

Complex	**1**		**2**
	Co1	Co2	Co1
Energies [cm^{-1}]	0 (d_{xy})	0 (d_{xy})	0 (d_{x2-y2})
	604.5 (d_{xz})	384.6 (d_{yz})	487.2 (d_{xz})
	1709.4 (d_{yz})	1765.4 (d_{xz})	1811.8 (d_{yz})
	6269.3 (d_{x2-y2})	6055.6 (d_{x2-y2})	6413.7 (d_{xy})
	10647.4 (d_{z2})	10754.9 (d_{z2})	10542.7 (d_{z2})

S-6. Single crystal X-Ray diffraction data

Table S3. Crystal data and structure refinement for **1** and **2**

Compounds	**1**	**2**
CCDC	2161285	2161284
Formula	$\{[Co_3Rb_6(cpdc)_6(H_2O)_{12}]{\cdot}6H_2O\}_n$	$[CoCs_2(cpdc)_2(H_2O)_6]_n$
Molecular formula, *M*	$C_{30}H_{60}Co_3O_{42}Rb_6$, 1782.39	$C_{10}H_{20}CoCs_2O_{14}$, 689.01
Temperature [K]	296.15	296.15
Crystal system, space group	Triclinic, $P\bar{1}$	monoclinic, $P2_1/c$
a [Å]	10.4873(3)	10.9378(5)
b [Å]	12.5920(4)	5.2648(2)
c [Å]	12.6360(5)	17.3362(8)
α [°]	78.0790(10)	
β [°]	66.7330(10)	100.972(2)
γ [°]	78.8820(10)	
V [$Å^3$]	1488.37(9)	980.06(7)
Z, density [g/cm^3]	1, 1.989	2, 2.335
μ [mm^{-1}]	5.804	4.602
F(000)	879	658
Crystal size	0.25×0.10×0.03	0.30×0.12×0.01
$\theta_{min-max}$ range [°]	2.211-31.290	4.377-30.594
Index ranges	$-14 \leq h \leq 15$,<br>$-14 \leq k \leq 17$,<br>$-18 \leq k \leq 17$	$-15 \leq h \leq 15$,<br>$-7 \leq k \leq 6$,<br>$-24 \leq k \leq 24$
Refl. collected/unique	20372/9363	11249/2967
Refl. [I > 2σ(I)]	5309	2724
R_{int}	0.0499	0.0274
Goodness-of-fit on F^2	0.986	1.144
Final R indices	$R_1 = 0.0490$, $wR_2 = 0.0933$	$R_1 = 0.0270$, $wR_2 = 0.0506$
R indices (all data)	$R_1 = 0.1116$, $wR_2 = 0.1114$	$R_1 = 0.0309$, $wR_2 = 0.0517$
Diff. peak/hole [e·$Å^{-3}$]	1.039/–1.096	0.744/–0.858

S-7. H-bonds

Table S4. H-bonds for **1** and **2**

//the analysis was done using PLATON software [A.L.Spek, Acta Cryst. 2009, D65, 148-155]//

D-H…A	D-H [Å]	H…A [Å]	D…A [Å]	D-H-A [°]	Symmetry index
Complex **1**					
O1W-H1Wa...O7	0.85	1.85	2.693(4)	173.6	-1+*x*, +*y*, +*z*
O1W-H2Wa...O4	0.85	1.89	2.664(4)	151.4	
O2W-H2Wa...O4	0.85	1.92	2.759(4)	169.0	1+*x*, +*y*, +*z*
O2W-H2Wb...O4	0.85	2.01	2.721(4)	140.9	-*x*, 1-*y*, 1-*z*
O3W-H3Wa...O8	0.96	1.76	2.669(4)	157.2	-*x*, 1-*y*, 1-*z*
O3W-H3Wb...O5Wa	0.90	2.04	2.775(4)	138.4	-*x*, 1-*y*, 1-*z*
O3W-H3Wb...O5Wb	0.90	1.91	2.807(18)	172.0	-*x*, 1-*y*, 1-*z*
O4W-H4Wa...O9W	0.85	2.14	2.831(7)	138.3	
O4W-H4Wb...O4	0.85	1.98	2.823(4)	169.9	1+*x*, +*y*, +*z*
O5Wa-H5Wa...O7	0.85	1.87	2.721(4)	179.6	-1+*x*, +*y*, +*z*
O5Wa-H5Wb...O7W	0.85	1.89	2.736(6)	179.5	
O6W-H6Wa...O8W	0.85	1.94	2.775(6)	167.7	+*x*, +*y*, 1+*z*
O6W-H6Wb...O11	0.85	1.93	2.754(4)	164.7	1+*x*, +*y*, +*z*
O7W-H7Wa...O3	0.85	1.93	2.755(6)	164.1	
O8W-H8Wa...O8	0.94	2.00	2.756(5)	135.7	
O9W-H9Wb...O3	0.85	1.84	2.678(6)	169.1	1-*x*, -*y*, 2-*z*
Complex **2**					
O1W-H1Wa...O2	0.81	1.978	2.769	164.1	
O1W-H1Wb...O2W	0.82	1.906	2.723	173.0	+*x*, 1/2-*y*, 1/2+*z*
O2W-H2Wa...O3	0.82	1.940	2.736	162.1	
O2W-H2Wb...O1	0.82	2.152	2.962	167.5	-*x*, 1/2+*y*, 1/2-*z*
O3W-H3Wa...O4	0.82	2.075	2.841	156.5	-*x*, 2-*y*, 1-*z*
O3W-H3Wb...O4	0.81	1.986	2.800	178.0	1+*x*, +*y*, +*z*
